# Genome analysis of *Desulfotomaculum gibsoniae* strain Groll^T^ a highly versatile Gram-positive sulfate-reducing bacterium

**DOI:** 10.4056/sigs.5209235

**Published:** 2014-03-20

**Authors:** Jan Kuever, Michael Visser, Claudia Loeffler, Matthias Boll, Petra Worm, Diana Z. Sousa, Caroline M. Plugge, Peter J. Schaap, Gerard Muyzer, Ines A.C. Pereira, Sofiya N. Parshina, Lynne A. Goodwin, Nikos C. Kyrpides, Janine Detter, Tanja Woyke, Patrick Chain, Karen W. Davenport, Manfred Rohde, Stefan Spring, Hans-Peter Klenk, Alfons J.M. Stams

**Affiliations:** 1 Department of Microbiology, Bremen Institute for Materials Testing, Bremen, Germany; 2 Wageningen University, Laboratory of Microbiology, Wageningen, The Netherlands; 3 Albert-Ludwigs-University Freiburg, Institute of Biology II, Freiburg, Germany; 4 Wageningen University, Laboratory of Systems and Synthetic Biology, Wageningen, The Netherlands; 5 Department of Aquatic Microbiology, Institute for Biodiversity and Ecosystem Dynamics, University of Amsterdam, Amsterdam, The Netherlands; 6 Instituto de Tecnologia Quimica e Biologica, Universidade Nova de Lisboa, Oeiras, Portugal; 7 Winogradsky Institute of Microbiology Russian Academy of Sciences, Moscow, Russia; 8 DOE Joint Genome Institute, Walnut Creek, California, USA; 9 Los Alamos National Laboratory, Bioscience Division, Los Alamos, New Mexico, USA; 10 HZI – Helmholtz Centre for Infection Research, Braunschweig, Germany; 11 Leibniz Institute DSMZ - German Collection of Microorganisms and Cell Cultures, Braunschweig, Germany; 12 University of Minho, Centre of Biological Engineering, Braga, Portugal

**Keywords:** spore-forming anaerobes, sulfate reduction, autotrophic, anaerobic degradation of aromatic compounds, complete oxidizer, *Peptococcaceae*, *Clostridiales*

## Abstract

*Desulfotomaculum gibsoniae* is a mesophilic member of the polyphyletic spore-forming genus *Desulfotomaculum* within the family *Peptococcaceae*. This bacterium was isolated from a freshwater ditch and is of interest because it can grow with a large variety of organic substrates, in particular several aromatic compounds, short-chain and medium-chain fatty acids, which are degraded completely to carbon dioxide coupled to the reduction of sulfate. It can grow autotrophically with H_2_ + CO_2_ and sulfate and slowly acetogenically with H_2_ + CO_2,_ formate or methoxylated aromatic compounds in the absence of sulfate. It does not require any vitamins for growth. Here, we describe the features of *D. gibsoniae* strain Groll^T^ together with the genome sequence and annotation. The chromosome has 4,855,529 bp organized in one circular contig and is the largest genome of all sequenced *Desulfotomaculum* spp. to date. A total of 4,666 candidate protein-encoding genes and 96 RNA genes were identified. Genes of the acetyl-CoA pathway, possibly involved in heterotrophic growth and in CO_2_ fixation during autotrophic growth, are present. The genome contains a large set of genes for the anaerobic transformation and degradation of aromatic compounds, which are lacking in the other sequenced *Desulfotomaculum* genomes.

## Introduction

*Desulfotomaculum gibsoniae* strain Groll^T^ (DSM 7213) is a mesophilic sulfate-reducing bacterium isolated from a freshwater ditch in Bremen, Northern Germany [1,2]. It grows with a wide range of substrates, including organic acids, such as medium-chain fatty acids, short-chain fatty acids, and several aromatic compounds [1]. These substrates are degraded to CO_2_ coupled to sulfate reduction. The strain is also able to grow autotrophically with H_2_/CO_2_ and sulfate, and is able to ferment pyruvate and crotonate. In the absence of sulfate, it grows slowly on H_2_/CO_2,_ formate, and methoxylated aromatic compounds. *D. gibsoniae* does not require vitamins for growth.

The genus *Desulfotomaculum* is a heterogeneous group of anaerobic spore-forming sulfate-reducing bacteria, with thermophilic, mesophilic, and psychrophilic members that grow at neutral or alkaline pH values [3]. Their cell wall stains Gram-negative, but the ultrastructure of the cell wall is characteristic of Gram-positive bacteria [4]. They are physiologically very diverse. In contrast to Gram-negative sulfate-reducing bacteria and closely related *Clostridia*, very little is known about their physiology, but members of this genus are known to play an important role in the carbon and sulfur cycle in diverse habitats.

The *Desulfotomaculum* genus is divided phylogenetically into different subgroups [1]. To get a thorough understanding of the evolutionary relationships of the different *Desulfotomaculum* subgroups and the physiology of the individual species, it is important to have genome sequence information. Here, we present a summary of the features of *D. gibsoniae* strain Groll^T^, together with the description of the complete genomic sequencing and annotation. A special emphasis is put on the ability of this strain to grow on a large variety of aromatic compounds and the responsible genes, and its capacity for acetogenic growth in the absence of sulfate.

## Classification and features

**Figure 1:**
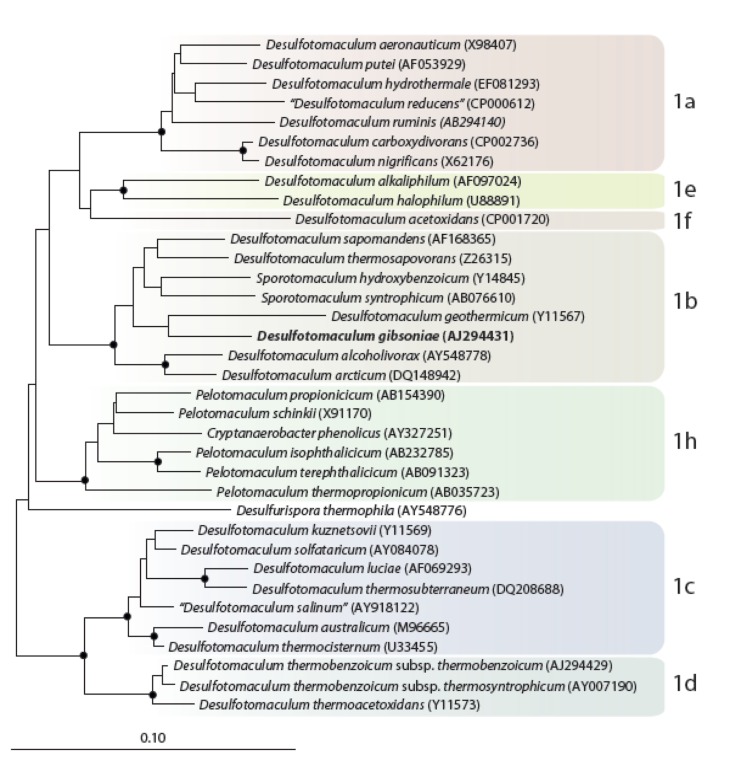
Neighbor joining tree based on 16S rRNA gene sequences showing the phylogenetic affiliations of *Desulfotomaculum* and related species and highlighted to show the subgroups of *Desulfotomaculum* cluster 1. *D. gibsoniae* is printed in bold type. The recently described *Desulfotomaculum defluvii* (cluster 1a), *Desulfotomaculum intricatum* (cluster 1f), *Desulfotomaculum peckii* (cluster 1e), and *Desulfotomaculum varum* (cluster 1a) and the entire cluster 1g are not included in the tree. A set of *Thermotogales* species were used as outgroup, but were pruned from the tree. Closed circles represent bootstrap values between 75 and 100%. The scale bar represents 10% sequence difference.

*D. gibsoniae* is a member of the phylum *Firmicutes*. Phylogenetic analysis of the 16S rRNA genes of *D. gibsoniae* shows that it clusters in *Desulfotomaculum* cluster 1, subgroup b. (Figure 1 [1]). Other species in this subgroup are *D. geothermicum, D. arcticum*, *D. alcoholivorax, D. thermosapovorans*, *D. sapomandens* and the non-*Desulfotomaculum* species *Sporotomaculum hydroxybenzoicum* and *S. syntrophicum*.

**Figure 2:**
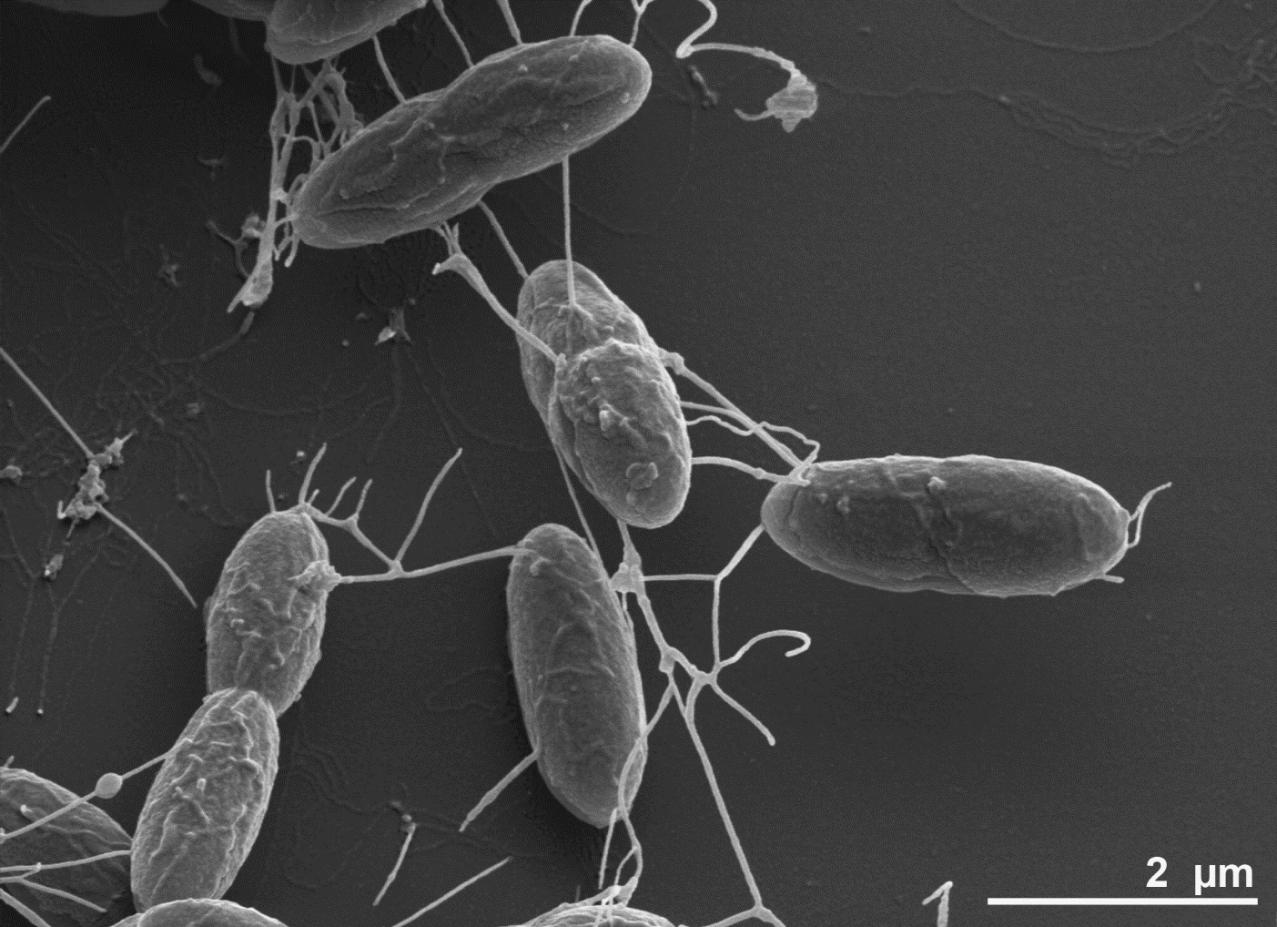
Scanning electron micrograph of *D. gibsoniae* strain Groll^T^.

*D. gibsoniae* is a mesophilic sulfate reducer, with an optimum growth temperature between 35-37°C [1,2]. Fermentative and acetogenic growth was shown with pyruvate, crotonate, formate, H_2_ + CO_2_, and methoxylated aromatic compounds as substrates. In the presence of an electron acceptor it can completely oxidize substrates to CO_2_. Suitable electron acceptors are sulfate, thiosulfate and sulfite. The cells of *D. gibsoniae* are straight or slightly curved rods (1.0-2.5 × 4-7 μm) with pointed ends (Figure 2). Spores of *D. gibsoniae* are spherical and located in the center of the cells, causing swelling. A summary of the classification and general features of *D. gibsoniae* is presented in Table 1.

**Table 1:** Classification and general features of *D. gibsoniae* strain Groll^T^ (DSM 7213) according to the MIGS recommendations [5].

MIGS ID	Property	Term	Evidence code^a^
	Current classification	Domain *Bacteria*	TAS [6]
		Phylum *Firmicutes*	TAS [6-8]
		Class *Clostridia*	TAS [9,10]
		Order *Clostridiales*	TAS [11,12]
		Family *Peptococcaceae*	TAS [11,13]
		Genus *Desulfotomaculum*	TAS [11,14,15]
		Species *Desulfotomaculum gibsoniae*	TAS [1]
		Type strain Groll	
	Gram stain	Negative with a Gram-positive cell wall structure	TAS [1,2]
	Cell shape	Straight or slightly curved rods with pointed ends	TAS [1,2]
	Motility	Motile, but motility was lost during cultivation	TAS [1,2]
	Sporulation	Spherical and central, slightly swelling the cell	TAS [1,2]
	Temperature range	20-40°C	TAS [1,2]
	Optimum temperature	35-37°C	TAS [1,2]
	Carbon source	CO_2_ (autotrophic) and many organic compounds including aromatic compounds	TAS [1,2]
	Energy source	Sulfate-dependent growth and fermentative growth with pyruvate, crotonate, formate, H_2_ + CO_2_, and methoxylated aromatic compounds	TAS [1,2]
	Electron acceptor	Sulfate, thiosulfate and sulfite	TAS [1,2]
MIGS-6	Habitat	Fresh water, mud, soil	TAS [1,2]
MIGS-6.2	pH range Optimum pH	6.0-8.0 6.9-7.2	TAS [1,2]
MIGS-6.3	Salinity	0-35 g l^-1^, no addition of NaCl necessary	TAS [1,2]
MIGS-22	Oxygen	Obligate anaerobe	TAS [1,2]
MIGS-15	Biotic relationship	Free living	TAS [1,2]
MIGS-14	Pathogenicity	BSF 1 [16]	
MIGS-4	Geographic location	Grolland, Bremen, Germany	TAS [1,2]
MIGS-5	Sample collection time	Spring 1989	
MIGS-4.1	Latitude	53.058 N	
MIGS-4.2	Longitude	8.762 E	
MIGS-4.3	Depth	60 cm (water), 1 cm sediment	

Evidence codes - TAS: Traceable Author Statement (i.e., a direct report exists in the literature); NAS: Non-traceable Author Statement (i.e., not directly observed for the living, isolated sample, but based on a generally accepted property for the species, or anecdotal evidence). Evidence codes are from the Gene Ontology project [17].

## Genome sequencing and annotation

### Genome project history

*D. gibsoniae* was selected for sequencing in the DOE Joint Genome Institute Community Sequencing Program 2009, proposal 300132_795700 'Exploring the genetic and physiological diversity of *Desulfotomaculum* species', because of its phylogenetic position in one of the *Desulfotomaculum* subgroups and its ability to use aromatic compounds for growth. The genome project is listed in the Genome OnLine Database (GOLD) [18] as project Gi07572, and the complete genome sequence is deposited in Genbank. Sequencing, finishing and annotation of the *D. gibsoniae* genome were performed by the DOE Joint Genome Institute (JGI) using state of the art sequencing technology [19]. A summary of the project information is shown in Table 2.

**Table 2:** Genome sequencing project information

**MIGS ID**	**Property**	**Term**
MIGS-31	Finishing quality	Finished
MIGS-28	Libraries used	Three genomic libraries: one Illumina shotgun library, one 454 standard library, and one paired end 454 library
MIGS-29	Sequencing platforms	Illumina GAii, 454 Titanium
MIGS-31.2	Genome coverage	479 × Illumina; 27.2 × pyrosequence
MIGS-30	Assemblers	Newbler v. 2.3
MIGS-32	Gene calling method	Prodigal, GenePRIMP
	INSDC ID	CP003273
	Genbank Date of Release	May 13, 2013
MIGS-13	GOLD ID NCBI project ID Database: IMG Source material identifier	Gc0017752 59873 2508501002 DSM 7213^T^
	Project relevance	Obtain insight into the phylogenetic and physiological diversity of *Desulfotomacum* species, and genes for anaerobic degradation of aromatic compounds

### Growth conditions and DNA isolation

*D. gibsoniae* strain Groll^T^, DSM 7213, was grown anaerobically in DSMZ medium 124a (*Desulfotomaculum* Groll Medium) [2,20] at 35°C. DNA was isolated from 0.5-1 g of cell paste using Jetflex Genomic DNA Purification kit (GENOMED 600100) following the standard protocol as recommended by the manufacturer. DNA quality was inspected according the guidelines of the genome sequence laboratory.

### Genome sequencing and assembly

The genome was sequenced using a combination of Illumina and 454 sequencing platforms. All general aspects of library construction and sequencing can be found at the JGI website [21]. Pyrosequencing reads were assembled using the Newbler assembler (Roche). The initial Newbler assembly consisting of 139 contigs in one scaffold was converted into a phrap [22] assembly by making fake reads from the consensus, to collect the read pairs in the 454 paired end library. Illumina GAii sequencing data (2,432 Mb) was assembled with Velvet [23] and the consensus sequences were shredded into 1.5 kb overlapped fake reads and assembled together with the 454 data. The 454 draft assembly was based on 220 Mb 454 draft data and all of the 454 paired end data. Newbler parameters are -consed -a 50 -l 350 -g -m -ml 21. The Phred/Phrap/Consed software package [22] was used for sequence assembly and quality assessment in the subsequent finishing process. After the shotgun stage, reads were assembled with parallel phrap (High Performance Software, LLC). Possible mis-assemblies were corrected with gapResolution [22], Dupfinisher [24], or sequencing cloned bridging PCR fragments with subcloning. Gaps between contigs were closed by editing in Consed, by PCR and by Bubble PCR primer walks (J.-F. Chang, unpublished). A total of 132 additional reactions were necessary to close some gaps and to raise the quality of the final contigs. Illumina reads were also used to correct potential base errors and increase consensus quality using a software Polisher developed at JGI [25]. The error rate of the final genome sequence is less than 1 in 100,000. Together, the combination of the Illumina and 454 sequencing platforms provided 506.2 × coverage of the genome. The final assembly is based on 2,347 Mb of Illumina draft data and 133 Mb of pyrosequence draft data.

### Genome annotation

Genes were identified using Prodigal [26] as part of the DOE-JGI genome annotation pipeline [27], followed by a round of manual curation using the JGI GenePRIMP pipeline [28]. The predicted CDSs were translated and used to search the National Center for Biotechnology Information (NCBI) non-redundant database, UniProt, TIGR-Fam, Pfam, PRIAM, KEGG, COG, and InterPro databases. Additional gene prediction analysis and functional annotation was performed within the Integrated Microbial Genomes - Expert Review (IMG-ER) platform [29].

## Genome properties

**Figure 3:**
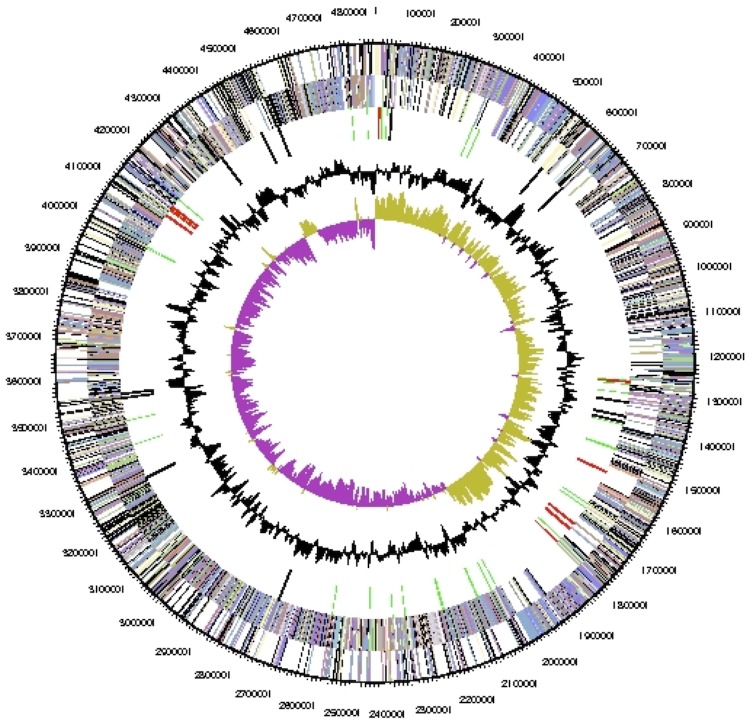
Graphical map of the chromosome. From outside to the center: Genes on forward strand (color by COG categories), genes on reverse strand (color by COG categories), RNA genes (tRNAs green, rRNAs red, other RNAs black), GC content (black), GC skew (purple/olive).

The genome consists of one circular chromosome of 4,855,529 bp (45.49% GC content) and includes no plasmids. A total of 4,762 genes were predicted, of which 4,666 are protein-coding genes. In addition, 3,464 of protein coding genes (72.7%) were assigned to a putative function with the remaining annotated as hypothetical proteins. The statistics of the genome are summarized in Table 3. 70.24% of the total genes were assigned to the COG functional categories (Table 4 and Figure 3).

**Table 3:** Genome statistics

**Attribute**	Value	% of total
Genome size (bp)	4,855,529	100.00
DNA coding region (bp)	3,949,133	81.33
DNA G+C content (bp)	2,208,827	45.49
Total genes	4,762	100.00
RNA genes	96	2.02
Protein-coding genes	4,666	97.98
Genes in paralog clusters	2,789	58.57
Genes assigned to COGs	3,345	70.24
Pseudo genes	314	6.59
Genes with signal peptides	737	15.48
Genes with transmembrane helices	966	20.29

**Table 4:** Number of genes associated with the general COG functional categories

**Code**	**Value**	**%age**	**Description**
J	157	4.26	Translation
A	1	0.03	RNA processing and modification
K	331	8.98	Transcription
L	276	7.49	Replication, recombination and repair
B	1	0.03	Chromatin structure and dynamics
D	51	1.38	Cell cycle control, mitosis and meiosis
Y	0	0.00	Nuclear structure
V	68	1.84	Defense mechanisms
T	237	6.43	Signal transduction mechanisms
M	169	4.58	Cell wall/membrane biogenesis
N	81	2.20	Cell motility
Z	0	0.00	Cytoskeleton
W	0	0.00	Extracellular structures
U	74	2.01	Intracellular trafficking and secretion
O	103	1.79	Posttranslational modification, protein turnover, chaperones
C	365	9.90	Energy production and conversion
G	104	2.82	Carbohydrate transport and metabolism
E	255	6.92	Amino acid transport and metabolism
F	73	1.98	Nucleotide transport and metabolism
H	188	5.10	Coenzyme transport and metabolism
I	158	4.29	Lipid transport and metabolism
P	151	4.10	Inorganic ion transport and metabolism
Q	75	2.03	Secondary metabolites biosynthesis, transport and catabolism
R	459	12.45	General function prediction only
S	309	8.38	Function unknown
-	1417	29.76	Not in COGs

## Insights into the genome

### Degradation of aromatic compounds

**Figure 4:**
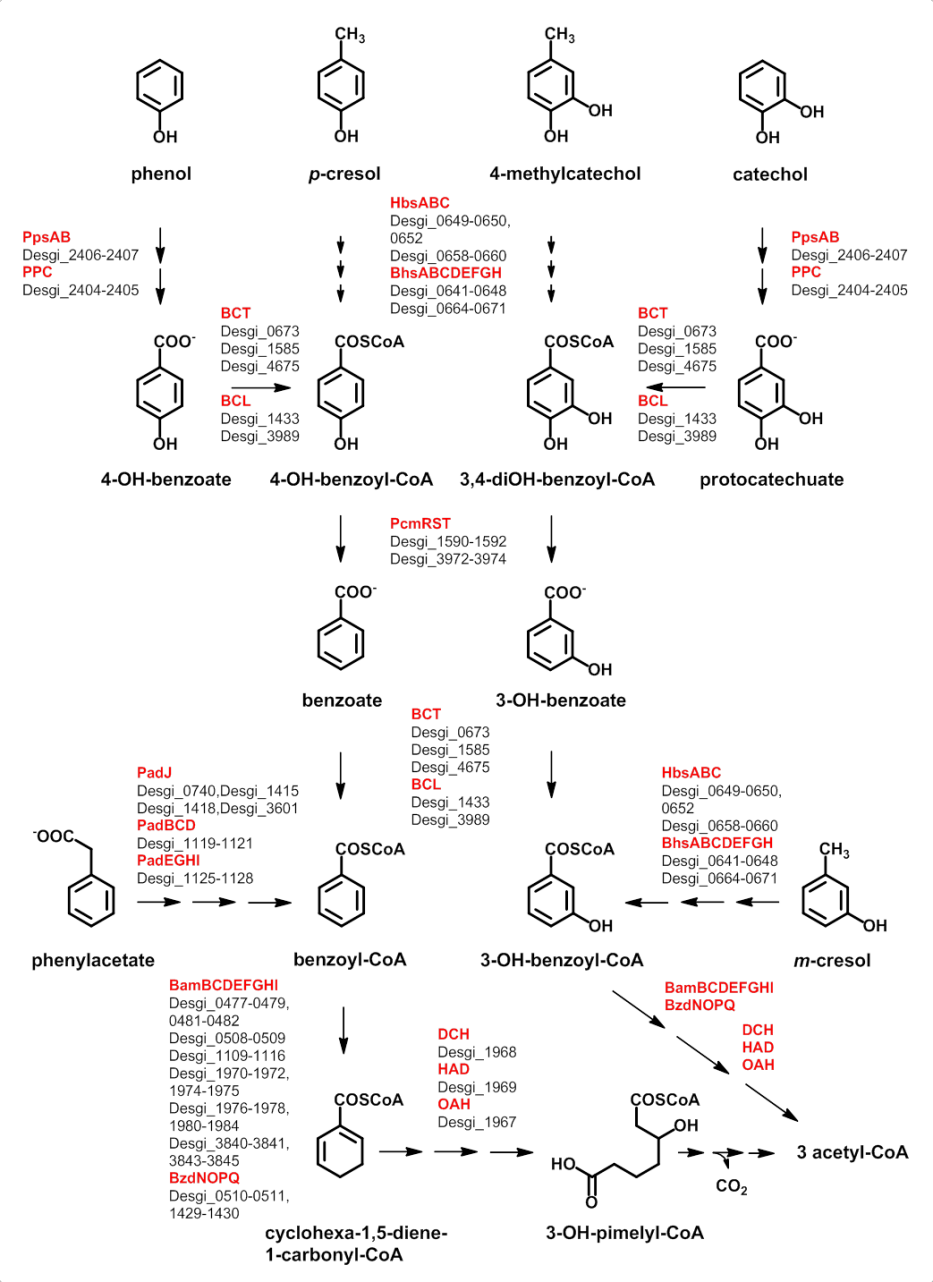
Degradation of aromatic compounds in *D. gibsoniae* based on genomic data. Enzymes are highlighted in red color. Possible encoding genes and their locus tags are shown below. Abbreviations: BamBCDEFGHI, class II benzoyl-CoA reductase; BCL, benzoate CoA-ligase; BCT, succinyl-CoA:benzoate CoA-transferase; BzdNOPQ, class I benzoyl-CoA reductase; BhsABCDEFGH, beta-oxidation of hydroxybenzylsuccinate; DCH, cyclohexa-1,5-diene-CoA hydratase; HAD, 6-OH-cyclohex-1-ene-1-carbonyl-CoA dehydrogenase; HbsABC, hydroxybenzylsuccinate synthase; OAH, 6-oxo-cyclohex-1-ene-1-carbonyl-CoA hydrolase; PadBCD, phenylacetyl-CoA acceptor oxidoreductase; padEGHI, phenylglyoxylate oxidoreductase; PadJ, phenylacetate CoA-ligase; PcmRST, 4-OH-benzoyl-CoA reductase; PPC, phenylphosphate carboxylase; PpsAB, phenylphosphate synthase.

*D. gibsoniae* can grow on a large variety of aromatic compounds (Figure 4) [1,2]. Other bacteria capable of growth via anaerobic degradation of aromatic compounds linked to nitrate reduction, Fe(III) reduction, or sulfate reduction are much more restricted [30,31].

In sulfate-reducing bacteria (e.g. *Desulfobacula toluolica*) methylated aromatic compounds such as toluenes, xylenes or cresols are thought to be degraded via an initial fumarate addition to the methyl group followed by β-oxidation-like reactions [32-34]. The genes putatively coding for the enzyme catalyzing the fumarate addition reaction (*hbs*ABC) are present in two copies in the genome of *D. gibsoniae*. They might have different substrate specificities for the growth substrates *m*- and *p*-cresol since the genome of *D. toluolica* possesses one set of these genes and can only grow with *p*-cresol [34,35]. In *D. gibsoniae* *m*- and *p*-cresol are expected to be converted to 3- or 4-hydroxybenzylsuccinate. The genes coding for enzymes involved in the subsequent β-oxidation (*bhs*ABCDEFGH), yielding 3- or 4-hydroxybenzoyl-CoA, are also present in two copies. In growth experiments toluene degradation was not observed for *D. gibsoniae* [1,2]. The genome provides no opposing information. All genes for the degradation of the growth substrates phenylacetate and phenol are present including the type of phenylphosphate carboxylase typically found in strict anaerobes [36].

All genes encoding enzymes of the upper benzoyl-CoA degradation pathway were identified in *D. gibsoniae*. The growth substrate benzoate is activated to benzoyl-CoA either via ATP-dependent CoA-ligase (*bcl*) or succinyl-CoA dependent CoA-transferase (*bct*) [37,38]. There are two classes of dearomatizing benzoyl-CoA reductases (BCRs) [39]. Class I are ATP-dependent FeS enzymes composed of four different subunits [40]. There are two subclasses of ATP-dependent BCRs of the *Thauera*- and the *Azoarcus*-type. ATP-independent class II BCRs contain eight subunits and harbor a tungsten-containing cofactor in the active site [41]. The ATP-independent class II BCR is characteristic of strictly anaerobic aromatic compound degrading bacteria [42]. In *D. gibsoniae* the genes of the catalytic subunit (*bam*B) of the class II BCR are present in six copies. All of the predicted seven genes for the putative electron activating subunits of class II BCR (*bam*CDEFGHI) were identified in at least two copies and arranged next to each other. Surprisingly, genes of a class I BCR with high similarity (47-68% amino acid identity) to class I BCRs of the *Azoarcus*-type (*bzd*NOPQ) were found, but these were not located in a single transcriptional unit. It is unclear which of the putative BCR-encoding genes is used for benzoyl-CoA and/or 3-OH-benzoyl-CoA reduction. The genes necessary to convert the product of BCRs, a cyclic conjugated dienoyl-CoA, to 3-OH-pimelyl-CoA via modified β-oxidation (*dch, had, oah*) are present in one copy each. It is unclear whether these genes are also involved in 3-OH-benzoyl-CoA degradation.

One of the more unusual growth substrates of *D. gibsoniae* is catechol, a substrate metabolized only by a very limited number of anaerobic bacteria. The pathway of catechol metabolism via protocatechuate was outlined 20 years ago [2] and is now confirmed by the genome analysis. For the degradation of lignin monomers, the side chains will be degraded and the methoxy-group will be removed by *o*-demethylation. The genes responsible for this mechanism are present in the genome (Desgi_0674 to Desgi_0676). The resulting compounds can then be degraded by the pathways outlined in Figure 4.

Phylogenetic trees based on *hbs*A which is a homolog to *bss*A (Figure 5A) and *hbs*C which is a homolog to*bss*C (Figure 5B) show deeply branching lineages for the *Desulfotomaculum gibsoniae* genes and no clear affiliation to other sulfate-reducing bacteria except, in the case of the *hbs*C gene to alkane-oxidizing species. Interestingly, similar genes were also found in the genomes of *Desulfotignum balticum* and *Desulfotignum phosphitoxidans*. Both are only known to use benzoate or its hydroxyl derivatives, whereas the only other species of this genus, *Desulfotignum toluenicum* can grow very well on toluene [44-46]. Using the *bam*B and *bam*C genes for phylogenetic tree construction (Figure 6A and Figure 6B), the picture is even more heterogenous. The different genes are affiliated with genes found in sulfate-reducing and other bacteria, hence a clear clustering cannot be seen. Again, genome data provides some interesting insights. *Desulfospira joergensenii* is not described as a benzoate utilizing bacterium, but seems to have some similar genes [47].

**Figure 5A:**
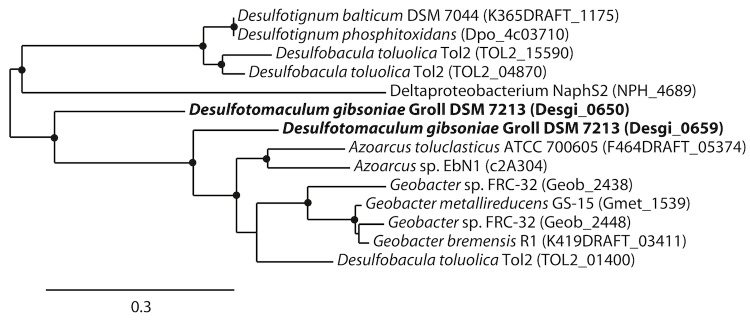
Phylogenetic tree based on amino acid sequences of *bssA* and *hbsA*. The trees were calculated with the "One-Click" mode of the online phylogenetic analysis program Phylogeny.fr [43]. Dots represent bootstrap values between 75 and 100%. The sequences of *Desulfotomaculum gibsoniae* are printed in bold. *Azoarcus* sp. EbN1 and *Geobacter* sp. FRC-32 are identical to *Aromatoleum aromaticum* and *Geobacter daltonii* respectively.

**Figure 5B:**
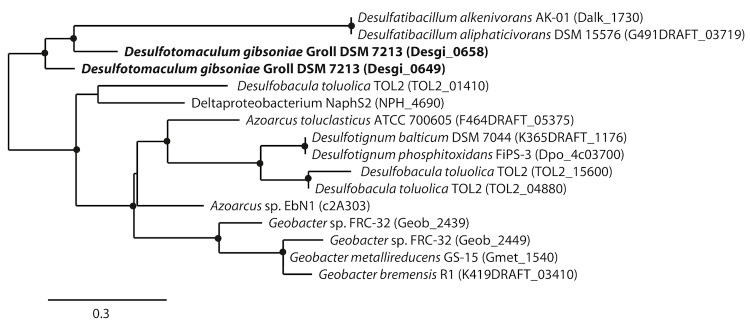
Phylogenetic tree based on amino acid sequences of *bssC* and *hbsC*. The trees were calculated with the "One-Click" mode of the online phylogenetic analysis program Phylogeny.fr [43]. Dots represent bootstrap values between 75 and 100%. The sequences of *Desulfotomaculum gibsoniae* are printed in bold. *Azoarcus* sp. EbN1 and *Geobacter* sp. FRC-32 are identical to *Aromatoleum aromaticum* and *Geobacter daltonii* respectively.

**Figure 6A:**
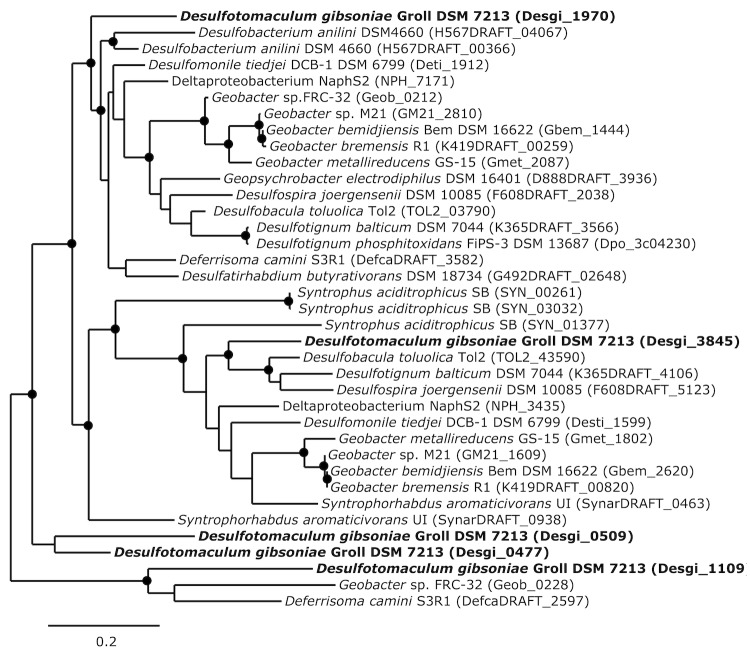
Phylogenetic tree based on amino acid sequences of five of the six homologs of *bamB*. The trees were calculated with the "One-Click" mode of the online phylogenetic analysis program Phylogeny.fr [43]. Dots represent bootstrap values between 75 and 100%. The sequences of *Desulfotomaculum gibsoniae* are printed in bold, *Geobacter* sp. FRC-32 is identical to *Geobacter daltonii*.

**Figure 6B:**
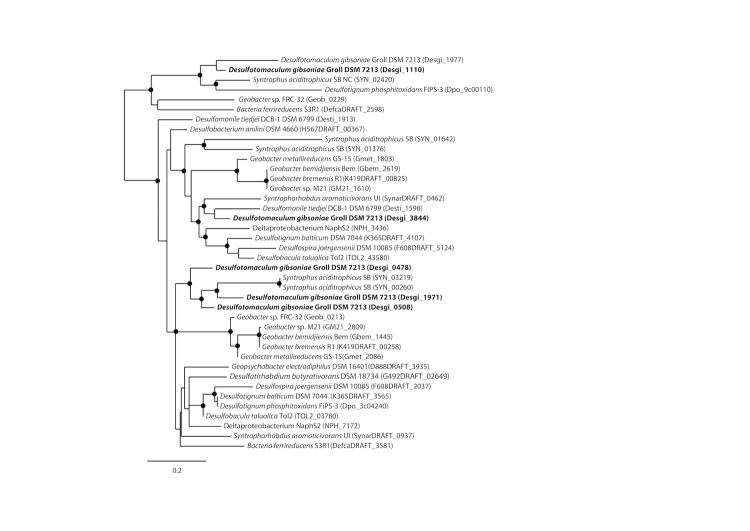
Phylogenetic tree based on amino acid sequences of six homologs of *bamC*. The trees were calculated with the "One-Click" mode of the online phylogenetic analysis program Phylogeny.fr [43]. Dots represent bootstrap values between 75 and 100%. The sequences of *Desulfotomaculum gibsoniae* are printed in bold, *Geobacter* sp. FRC-32 is identical to *Geobacter daltonii*.

### Complete substrate oxidation, autotrophic growth and homoacetogenic growth

**Figure 7:**
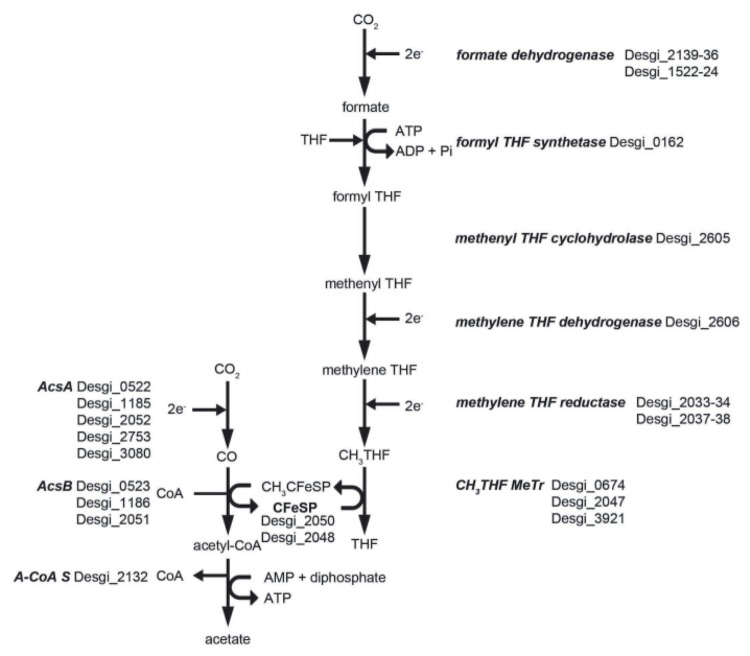
Acetyl-CoA pathway in *D. gibsoniae* based on genomic data. Enzymes are depicted in bold italic. Next to these enzymes are the possible encoding genes, and their locus tags. Genes with the locus tags Desgi_2048 and Desgi_2050 putatively code for the small subunit and the large subunit of the iron-sulfur protein, respectively. This protein is involved in transferring the methyl from tetrahydrofolate to acetyl-CoA. Abbreviations: A-CoA S, acetyl-CoA synthetase; AcsA, carbon monoxide dehydrogenase; AcsB, acetyl-CoA synthase; CFeSP, iron-sulfur protein; CH_3_, methyl; THF, tetrahydrofolate; MeTr, methyltransferase.

The genome of *D. gibsoniae* contains putative genes that code for the enzymes of the complete tricarboxylic acid (TCA) cycle: Citrate synthase, Desgi_1296, 2412; aconitase, Desgi_1576; isocitrate dehydrogenase, Desgi_4665; 2-oxoacid:ferredoxin oxidoreductase, Desgi_0085-0088, 2095, 2585-2588, 3041-3044; succinyl-CoA synthetase, Desgi_1954-1955; succinate dehydrogenase, Desgi_0077-0080, 3996-3998; fumarase, Desgi_0075, 1952-1953; malate dehydrogenase, Desgi_1960. These genes could be involved in the complete oxidation to CO_2_ by *D. gibsoniae*. Moreover, the complete acetyl-CoA pathway is also present in the genome of *D. gibsoniae* (Figure 7). However, *D. gibsoniae* is not able to grow on acetate with or without sulfate. The acetyl-CoA pathway in *D. gibsoniae* does not perform acetate oxidation, as described in *D. kuznetsovii* [48], but facilitates complete oxidation of substrates leading to acetyl-CoA, autotrophic growth on H_2_ + CO_2_ (or formate) in the presence of sulfate as electron acceptor, and slow homoacetogenic growth on pyruvate, crotonate, formate, hydrogen plus carbon dioxide, and methoxylated aromatic compounds [1].

**Figure 8:**
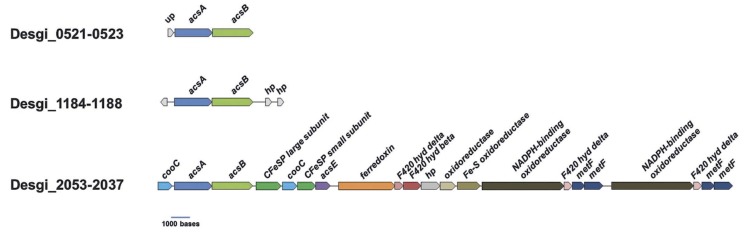
Gene orientation of putative coding acetyl-CoA synthase and neighboring genes in the genome of *D. gibsoniae*. Abbreviations: acsA, carbon monoxide dehydrogenase; acsB, acetyl-CoA synthase; acsE, methyl-tetrahydrofolate methyltransferase; CFeSP, iron-sulfur protein; cooC, carbon monoxide dehydrogenase maturation factor; Fe-S, Iron sulfur; hp, hypothetical protein; hyd, hydrogenase; metF, methylene-tetrahydrofolate reductase; up, uncharacterized protein.

Three putative acetyl-CoA synthase encoding genes can be found in the *D. gibsoniae* genome (Figure 8). All three genes have a putative carbon monoxide dehydrogenase catalytic subunit encoding gene (*coo*S) downstream. However, only Desgi_2051 is part of an operon structure containing other genes coding for enzymes involved in the acetyl-CoA pathway.

### C1 compound degradation

In addition to the three *coo*S genes downstream of the genes coding for the acetyl-CoA synthase, *D. gibsoniae* has two other *coo*S genes in its genome, Desgi_2753, and Desgi_3080. The latter has a transcriptional regulator (Desgi_3081) downstream and a ferredoxin (Desgi_3079) and a nitrite reductase (Desgi_3078) upstream. Growth tests on CO have not yet been performed. However, the presence of multiple *coo*S genes with neighbor genes like ferredoxin and nitrate reductase, or genes coding for the acetyl-CoA pathway indicates that *D. gibsoniae* may grow on CO.

*D. gibsoniae* can grow on formate coupled to sulfate reduction. In the genome, two putative formate dehydrogenases (FDHs) were found. One FDH (Desgi_1522-23) is translocated over the membrane and bound to a polysulfide reductase (NrfD)-like protein containing 10 trans-membrane helixes (Desgi_1524). The alpha subunit contains a twin-arginine translocation (tat) motif and genes encoding proteins of the Tat system; TatA (Desgi_1521) and TatC (Desgi_1526) were found near the alpha subunit coding gene. The second FDH (Desgi_2136-2139) might be a confurcating FDH. Desgi_2138 shows similarity with the NADH binding 51kD subunit of NADH:ubiquinone oxidoreductase and Fe-S cluster binding motifs, which were found in all subunits.

No methanol methyltransferase genes can be found in the genome of *D. gibsoniae*, which correlates with the absence of growth on methanol [1]. Other methyltransferase genes that might point to growth with methylated amines were not found, except for a possible dimethylamine methyltransferase beta subunit (Desgi_3904) and a cobalamin binding protein (Desgi_3903). However, another methyltransferase gene, *mtb*A, which is absent from the genome, is necessary for growth with dimethylamine.

### Propionate and butyrate oxidation

**Figure 9:**
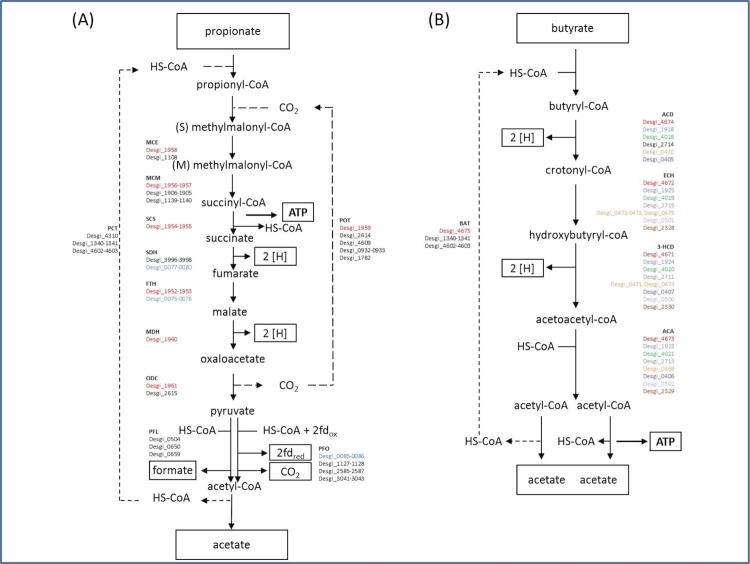
(A) Propionate and (B) butyrate degradation pathways in *D. gibsoniae* based on genomic data. The two main gene clusters for propionate and butyrate degradation are indicated in red and blue colors. For butyrate degradation, additional gene clusters were found, shown in different colors. For the other steps in the propionate degradation pathway, genes were found to be located in different places in the genome. Abbreviations: PCT, propionate CoA transferase; MCE, methylmalonyl-CoA epimerase; MCM, methylmalonyl-CoA mutase; SCS, succinyl-CoA synthase; SDH, succinate dehydrogenase; FHT, fumarase (fumarate hydratase); MDH, malate dehydrogenase; ODC, oxaloacetate decarboxylase; PFO, pyruvate:ferredoxin oxidoreductase; PFL, pyruvate formate lyase; POT, propionyl-CoA:oxaloacetate transcarboxylase; BAT, butyryl-CoA:acetate-CoA transferase; ACD, acyl-CoA dehydrogenase; ECH, enoyl-CoA hydratase; 3-HCD, 3-hydroxybutyryl-CoA dehydrogenase; ACA, acetyl-CoA acetyltransferase.

The genome of *D. gibsoniae* contains at least one copy of genes putatively encoding enzymes involved in propionate oxidation via the methylmalonyl-CoA pathway (Figure 9A). This includes genes in a methylmalonyl-CoA (*mmc*) cluster (Desgi_1951-1961), which have a genetic organization similar to those seen *D. kuznetzovii* (Desku_1358-1369) and *Pelotomaculum thermopropionicum* (Pth_1355-1368) [48-50]. However, a few differences were found. The genome of *D. gibsoniae* lacks genes coding for methylmalonyl-CoA decarboxylase epsilon and gamma subunits. Moreover, the *mmc* cluster of *D. gibsoniae* contains a single gene encoding the alpha subunit of succinyl-CoA synthase (Desgi_1955), whereas the *mmc* clusters of *D. kuznetzovii* and *P. thermopropionicum* contain two encoding genes. Bifurcating hydrogenases may be used to re-oxidize ferredoxin, which is generated by pyruvate:ferredoxin oxidoreductase and NADH, which in turn is generated from malate dehydrogenation for the formation of hydrogen. The membrane-anchored extracellular formate dehydrogenases and hydrogenases may be involved in generating a proton motive force for succinate reduction.

**Figure 10:**
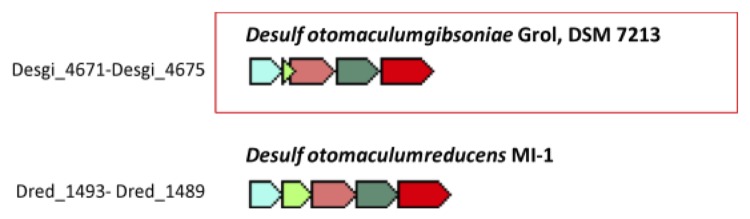
Orthologous neighborhood genes for gene cluster Desgi_4671- 4675, encoding enzymes necessary for butyrate degradation. Light blue – 3-hydroxybutyryl-CoA dehydrogenase (3-HCD); green – enoyl-CoA hydratase (ECH); old pink – acetyl-CoA acetyltransferase (ACA); dark green – Acyl-CoA dehydrogenase (ACD); red – butyryl-CoA:acetate CoA-transferase (BAT).

**Figure 11:**
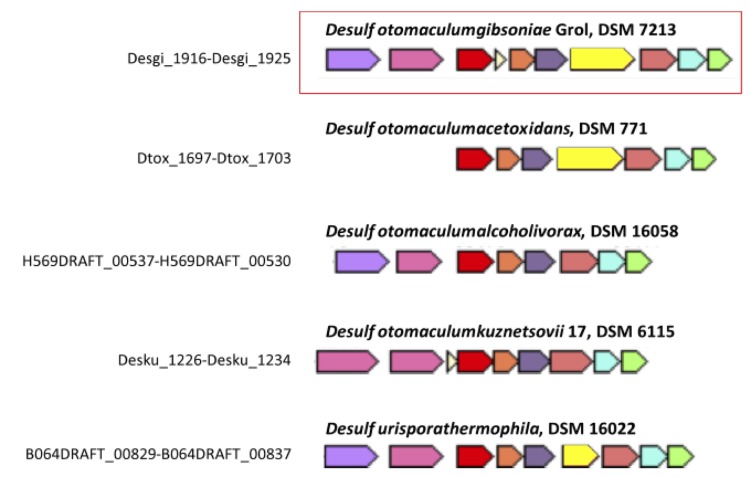
Orthologous neighborhood genes for gene cluster Desgi_1916- 1925, encoding enzymes necessary for butyrate degradation. Purple/pink – acyl-CoA synthetase; red – acyl-CoA dehydrogenase (ACD); orange/dark purple – electron transfer flavoprotein; yellow – Fe-S oxidoreductase; old pink – acetyl-CoA acetyltransferase (ACA); light blue – 3-hydroxybutyryl-CoA dehydrogenase (3-HCD); green – enoyl-CoA hydratase (ECH).

Genes putatively coding for butyrate β-oxidation enzymes are also present in the genome of *D. gibsoniae*. One complete cluster of genes putatively encoding all the enzymes required to convert butyrate is present (Desgi_4671-4675, Figure 9B). Gene organization in this cluster is similar to that found in *D. reducens* (Dred_1493-1489), which can also utilize butyrate (Figure 10A). In *D. gibsoniae* another gene cluster (Desgi_1916-1925) is present which only lacks one gene coding for butyryl-CoA:acetate CoA-transferase (Figure 11). Desgi_1918 and Desgi_1920-1925 have a similar organization to genes found in *D. acetoxidans* (Dtox_1697-1703) [51]. In addition to the genes encoding enzymes involved in butyrate β-oxidation, these clusters contain genes for electron transfer flavoproteins (Desgi_1920-1921 and Dtox_1698-1699) and for Fe-S oxidoreductases (Desgi_1922 and Dtox_1700). Although Dtox_1700 is annotated as a cysteine-rich unknown protein, a protein blast of these ORFs against the *D. gibsoniae* genome revealed 53.65% identity (Evalue = 0.0) with the putative Fe-S oxidoreductase encoded by Desgi_1922. Two genes encoding acyl-CoA synthetases (Desgi_1916-1917) are present upstream of the acetyl-CoA dehydrogenase gene in *D. gibsoniae* (Desgi_1918), but these are not found near this cluster in *D. acetoxidans*. However, these genes are present in the same gene cluster location in other butyrate-degrading sulfate-reducing bacteria (SRB), namely *D. alcoholivorax* (H569DRAFT_00537-00530), *D. kuznetsovii* (Desku_1226-1234) and *Desulfurispora thermophila* (B064DRAFT_00829-00837). Acyl-CoA synthetases are most likely involved in the biosynthesis of coenzyme A [52]. Several other clusters of genes containing at least three genes encoding enzymes involved in butyrate conversion can be found in the genome of *D. gibsoniae*.

### Sulfate reduction

The genome contains single copies of the sulfate adenyltransferase (Desgi_3703), adenosine-5´-phosphosulfate (APS) reductase (Desgi_3701–3702) and dissimilatory sulfite reductase (Desgi 4661-4662) as are found in most of the other members of the genus [18-20]. A membrane-bound pyrophosphatase (Desgi_4294) is used for energy regeneration as in other *Desulfotomaculum* spp. The QmoABC complex contains only the A and B subunit, the C subunit is lacking (Desgi_3699–3700). In all members of the genus *Desulfotomaculum* the QmoAB is followed by HdrCB (Desgi_3697–3698). This arrangement is identical to that seen in the closely related species “*Desulforhudis audaxivator*”, *Desulfurispora thermophila* and the Gram-negative *Desulfarculus baarsii* and strain NaphS2, which possess a Gram-positive AprBA [53]. Interestingly, the same organization is also found in some phototrophic sulfur-oxidizing bacteria, such as *Thiobacillus dentrificans*, *Thiothrix nivea* and *Sedimentibacter selenatireducens* [54]. Other closely related Gram-positive SRB like *Desulfovirgula thermoconiculi* and *Ammonifex degensii* have a complete QmoABC system like all other SRB and the Green Sulfur Bacteria, or have QmoAB linked to a Fe-S oxidoreductase/HdrD as seen in *Desulfosporosinus* spp. This latter modification is also seen in other Gram-negative SRB, which have a Gram-positive AprBA-like *Desulfomonile tiedjei* and *Syntrophomonas fumaroxidans* [55]. It seems that both *Desulfotomaculum* sp. and *Desulfosporosinus* have been the source of the entire aps reductase/ QmoA complex for members of the Gram-negative *Syntrophobacterales* [55]. The genomes of *Syntrophobacter fumaroxidans* and of *Desulfovirgula thermoconiculi* have two different systems that can be linked to the aps reductase.

In *D. gibsoniae* the *dsr*AB (Desgi_4661–4662) is linked to the same truncated *dsr* operon coding only for *dsr*C and *dsr*MK (Desgi 4648–4649) as in other *Desulfotomaculum* spp [48,51,56].

### Hydrogenases

*D. gibsoniae* has six [FeFe] and three [NiFe] hydrogenases, suggesting a lower redundancy in the case of [FeFe] enzymes than other members of the genus. The [FeFe] hydrogenases include one membrane-associated protein (Desgi_0926-0928) that contains a tat motif in the alpha subunit (Desgi_0926), suggesting an extracellular localization; one monomeric hydrogenase (Desgi_0935) encoded close to the membrane-bound enzyme, which suggests the possibility of co-regulation; two copies of trimeric NAD(P)-dependent bifurcating hydrogenases (Desgi_4669-4667 and Desgi_3197-3195); one enzyme (Desgi_0771) that is part of a multi-gene cluster encoding two flavin-dependent oxidoreductases that is also present in other *Desulfotomaculum* spp., and one HsfB-type hydrogenase (Desgi_3194) encoding a PAS-sensing domain that is likely involved in sensing and regulation, and possibly with the bifurcating Desgi_3195 hydrogenase.

The [NiFe] hydrogenases include one enzyme (Desgi_1398 – 1397) that may also be bound to the membrane by a cytochrome b (Desgi_1402); one simple dimeric enzyme (Desgi_1231-1230); and one trimeric group 3 hydrogenase (Desgi_1166-1164), similar to methyl-viologen reducing hydrogenases from methanogens, and which is encoded next to a HdrA-like protein (Desgi_1163).

### Nitrogenases

A cluster of nitrogenase genes, specifically genes encoding nitrogenase iron protein, nitrogen regulatory protein PII, nitrogenase molybdenum-iron protein alpha chain, nitrogenase molybdenum-iron protein beta chain, nitrogenase molybdenum-iron cofactor biosynthesis protein *Nif*E, nitrogenase molybdenum-iron protein, alpha and beta chains, nitrogenase cofactor biosynthesis protein *Nif*B; ferredoxin, iron only nitrogenase protein *Anf*O (*Anf*O_nitrog) (Desgi_2428-2419) were detected within the annotated genome sequence. Thus, *D. gibsoniae* probably has the capacity for nitrogen fixation. However, the fixation of molecular nitrogen has not been analyzed in this species so far.
